# Outpatient Treatment of Mild Dysphagia in Ear-Nose-and-Throat Clinics

**DOI:** 10.7759/cureus.52395

**Published:** 2024-01-16

**Authors:** Takafumi Yamano, Kensuke Nishi, Fumitaka Omori, Ayumi Nakamura, Kazumasa Fukuyo

**Affiliations:** 1 Department of Otorhinolaryngology, Department of Medicine, Fukuoka Dental College, Fukuoka, JPN; 2 Department of Otorhinolaryngology, Fukuoka Dental College Hospital, Fukuoka, JPN; 3 Department of Physical Medicine and Rehabilitation, Fukuyo ENT Clinic, Itoshima, JPN; 4 Department of Otorhinolaryngology, Fukuyo ENT Clinic, Itoshima, JPN

**Keywords:** fibreoptic endoscopic evaluation of swallowing (fees), swallowing, outpatient, ent clinic, dysphagia

## Abstract

Objective: Many reports on inpatient dysphagia rehabilitation in acute and convalescent rehabilitation hospitals exist, but there are a few reports on outpatient treatments. Otolaryngologists still take a trial-and-error approach when treating dysphagia. Here, we explore the effectiveness and limitations of outpatient treatment in ear-nose-and-throat (ENT) clinics.

Methods: Sixty-four patients (41 males and 23 females) aged 27-101 years (mean 78 years) visited an outpatient clinic specialising in feeding and swallowing conditions (the Fukuyo ENT Clinic). All were able to perform the activities of daily living (ADL) to the extent that outpatient visits were possible; no home visits were made. The weekly outpatient day was staffed by an otolaryngologist and a speech-language-hearing therapist (SLHT). All patients were subjected to fibreoptic endoscopic evaluation of swallowing (FEES), followed by appropriate training as revealed by the examinations.

Results: Salivary retention in the glottis valley and piriform sinuses improved (both p < 0.05) in 30 patients who underwent repeat FEES; we compared the initial and final figures. In 14 cases in whom maximal tongue pressure (TP) was measured, this was higher at the final than at the first examination (p < 0.01).

Conclusion: Outpatient treatment at ENT clinics for patients who are able to maintain their ADLs to the extent that they are able to walk to a hospital is an option for the treatment of age-related dysphagia. For severe cases, however, house calls and collaboration with the home and nursing care sector will be necessary and should be considered in the future.

## Introduction

Many reports on inpatient dysphagia rehabilitation in acute and convalescent hospitals exist, but there are a few reports on outpatient treatment. Many patients experience difficulties with the activities of daily living (ADL), and speech-language-hearing therapists (SLHTs) who train patients are only a few. Inevitably, inpatient treatment is the principal focus; outpatient treatment is provided by few facilities. Otolaryngologists take a trial-and-error approach when treating dysphagia, and treatments differ by facility; treatments may be delivered during normal clinic hours or via house calls in Japan.

Furthermore, it is not uncommon for older adults to develop delirium after being admitted to hospital; risk factors include a history of dementia, cognitive impairment, functional impairment, alcohol use disorder, age over 70 years, high prevalence of dependence, and comorbidities such as cerebrovascular disease and depression [1]. These factors are more common in older people with dysphagia. Outpatient treatment avoids the aforementioned risks, but the severity of illness, frequency of visits, interventions, and assessment methods vary according to the situation at each facility, and no guidelines exist. In addition, the involvement of otolaryngologists in swallowing disorders is still evolving.

We provide outpatient treatment, mainly for minor cases, and few studies have developed protocols and interventions based on objective fibreoptic endoscopic evaluation of swallowing (FEES) and video fluorography (VF) assessment.

The Fukuyo ENT Clinic, Fukuoka, Japan, provides outpatient treatments for mild dysphagia. We evaluated the effectiveness and limitations of outpatient dysphagia treatment in ENT clinics and the role of ENT in swallowing care.

## Materials and methods

Patients

**Figure 1:**
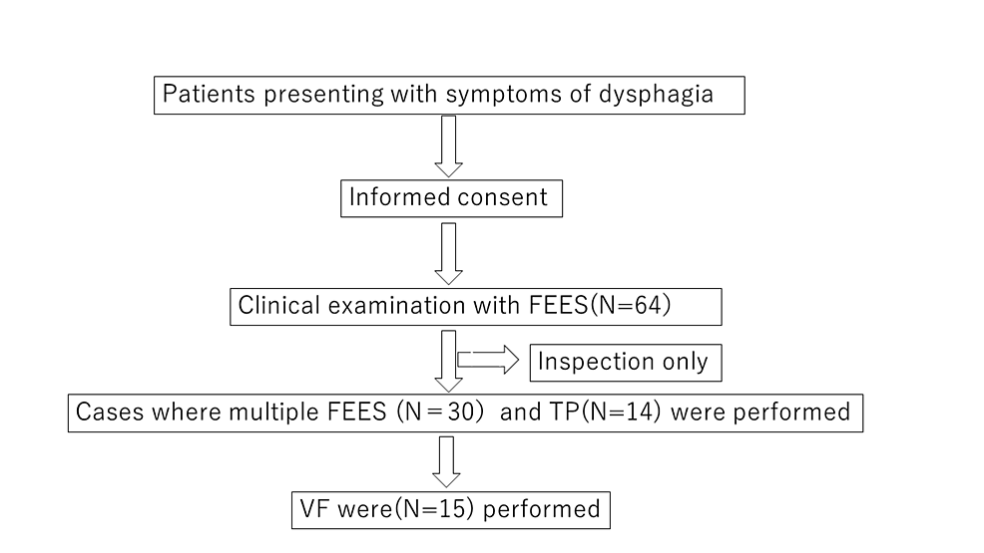
Flow chart of patient selection.

Sixty-four patients (41 males and 23 females) aged between 27 and 101 years (average 78 years) visited an outpatient clinic that specialised in feeding and swallowing issues (the Fukuyo ENT Clinic) between September 2020 and December 2022. All subjects could perform ADL to the extent that outpatient visits were possible; no house calls were made. In addition, the interview confirmed the absence of gastric acid reflux or any medications that could affect swallowing function. A flowchart of patient selection is shown in Figure 1. Of the 64 patients, 34 underwent a single examination, and 30 had an outpatient visit and multiple FEES. Among them, 14 had tongue pressure (TP), and 15 had VF measured.

Clinical structure

The once-weekly outpatient days were staffed by one otolaryngologist and one SLHT. On the first visit, the otolaryngologist examined the pharynx to exclude tumours and other organic conditions and performed FEES. The SLHT provided general guidance to patients, including instructions on eating posture and appropriate food morphologies, food suggestions, swallowing exercises, and oral care. Commencing at the second visit, the otolaryngologist conducted a medical interview and performed FEES at about monthly intervals. The SLHT continued patient training, informed by the FEES findings. The visit frequency was generally weekly but was decided via consultation between the staff and each patient’s family.

Swallowing function

The FESS results were evaluated using the Hyodo scoring system, [2] which explores four parameters: salivary pooling in the vallecular and piriform sinuses; induction of the glottal closure reflex on touching the epiglottis or arytenoid with the endoscope; initiation of the swallowing reflex as assessed by the “whiteout” time, thus the period during which the endoscopic image is obscured by pharyngeal closure; and the extent of pharyngeal clearance after swallowing 3 mL of blue-dyed water. All parameters were scored using a 4-point scale (0: normal; 1: mildly impaired; 2: moderately impaired; 3: severely impaired), with higher scores indicating greater swallowing dysfunction.

The indications for VF were unclear FEES data; suspected oral phase disorders, such as feeding/oral retention; suspected oesophageal phase disorders, such as impaired passage and/or reflux; and expected difficulties with continued oral intake. VF was evaluated using a described method [3]. Briefly, while in a sitting or semi-sitting position, the patient was asked to swallow 10 mL of non-ionic iodinated contrast agent (Omnipaque 300®) for a single examination.

Tongue pressure (TP)

A JM-TPM02E instrument (JMS Co., Ltd. Hiroshima, Japan) was used to measure the maximal TP. Two measurements were taken, and the average was calculated. Indications for TP measurement included tongue atrophy on examination of the oral cavity or suspected oral phase disorders such as early pharyngeal inflow evident on FEES or disorders in terms of residual oral material or food apparent on VF.

Rehabilitation

The rehabilitation protocol has been described previously [4] and included indirect and direct training, oral care, instruction on appropriate food morphology, eating methods, compensatory swallowing modes, respiratory expectoration (abdominal breathing, huffing, and vocal training), oral swallowing and articulation, environmental conditioning, and physical fitness. From the FEES findings, the necessary menu was selected, and one rehabilitation session was set to last approximately 30 minutes. Exercises that could be performed at home were actively encouraged.

Data analysis

The McNemar test was used to compare the initial and final FEES scores, and the paired t-test was employed to compare the TPs. The study was approved by the Fukuoka Academy Research Ethics Committee (permit no. 628). Written informed consent was obtained from all patients or their guardians.

## Results

Causes of dysphagia

Ageing was the cause in 39 (61%) cases, cerebrovascular disease in eight (13%), dementia in six (9%), the sequelae of head and neck cancer treatment in six (9%), the sequelae of gastric cancer treatment in three (5%), and degenerative diseases in two (3%) cases.

The complaints

Pharyngeal discomfort was the most common complaint (28 cases, 44%), followed by swallowing problems (21 cases, 33%), dysphagia (seven cases, 11%), difficulties when taking medication (five cases, 8%), cough (two cases, 3%), and prolonged mealtimes (one case, 1%) (Table 1).

**Table 1:** The principal complaints.

Main complaint	Number	％
Discomfort	28	44
Choking	21	33
Sensation of difficulty in swallowing	7	11
Difficulty in taking medication	5	8
Cough	2	3
Extended meal times	1	1

Initial FEES scores

Forty cases exhibited initial FEES scores of 0-4 (62%) and 24 scores of 5-8 (38%). No case evidenced a score ≥ 9, which would have indicated that oral intake was difficult (Table 2).

**Table 2:** The initial FEES scores.

FEES score	Number	％
0-4 points	40	62
5-8 points	24	38
9 and above	0	0

Duration of outpatient visits

Including cases who underwent assessment only, 30 attended for less than 30 days, nine for 90-180 days, and 11 for more than 180 days (Table 3).

**Table 3:** Duration of outpatient visits.

Length of hospital stay	Number	％
Less than 30 days	30	45
More than 30 days but less than 90 days	17	25
More than 90 days but less than 180 days	9	14
More than 180 days	11	16

Comparison of the initial and final itemised FEES scores

**Figure 2:**
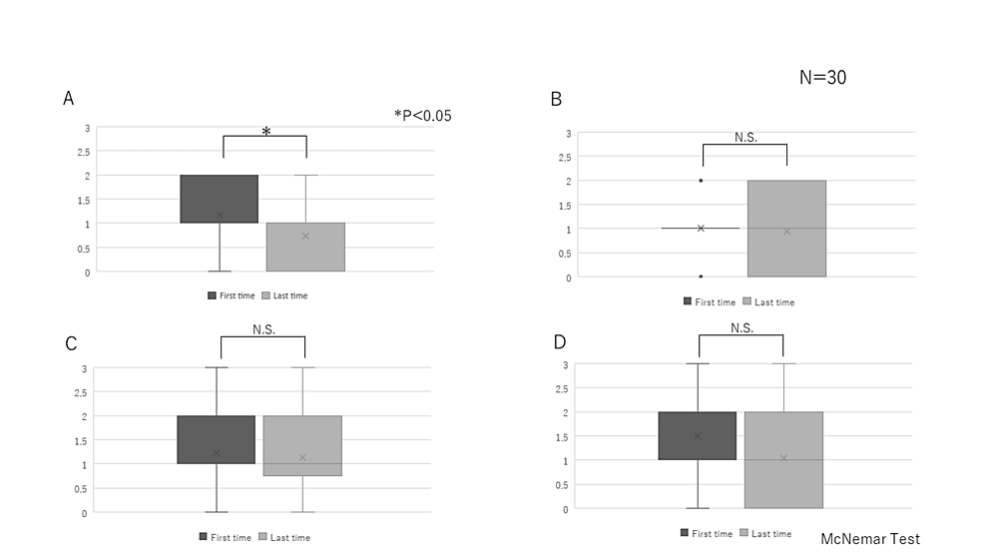
Comparisons of the first and final FEES scores. In 30 cases, multiple assessments were conducted. The McNemar test was used. A.　The extent of salivary pooling in the vallecular and piriform sinuses (＊p＜0.05). B.　The glottal closure reflex induced by touching the epiglottis or arytenoid with the endoscope. C.　The location of the bolus at initiation of the swallowing reflex as assessed by the “white-out” time. D.　The extent of pharyngeal clearance after swallowing of blue-dyed water.

In 30 cases, multiple assessments were conducted. The extent of salivary pooling in the vallecular and piriform sinuses improved (Figure 2A). No significant difference over time was apparent in the glottal closure reflex induced by touching the epiglottis or arytenoid with the endoscope. The bolus location at the time of swallowing reflex initiation was assessed by determining the “white-out” period and did not change over time. The extent of pharyngeal clearance after swallowing of blue-dyed water also did not change over time (Figures 2B-2D).

Comparison of the initial and final maximal TP

**Figure 3:**
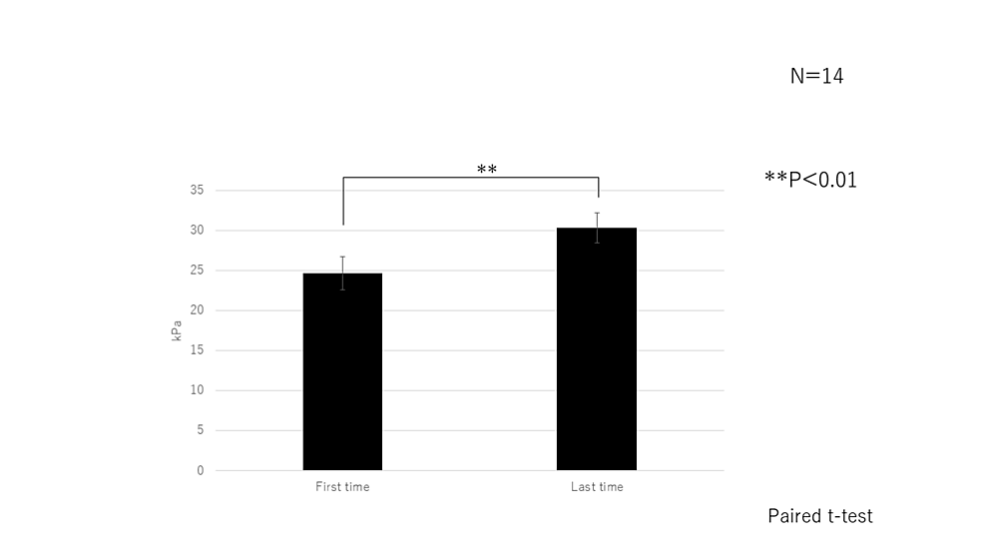
Comparisons of the initial and final maximal tongue pressures. The final maximal pressures were higher (**p＜0.01) of 14 patients. The paired t-test was employed.

In 14 cases, multiple assessments were performed. The maximum TP increased over time (Figure 3).

Details of the 15 patients who underwent VF

Patient ages ranged from 27 to 87 years; 11 were male and four were female. The FEES scores ranged from 0 to 4 in four cases and from 5 to 8 in 11 cases; a higher proportion of patients had elevated scores. The duration of hospital visits was less than 30 days in three cases, from 30 to 90 days in five, from 90 to 180 days in two, and more than 180 days in five; most patients had long histories of hospital visits. On examination, oral and oesophageal disorders were found in two cases, and aspiration was confirmed in eight (Table 4).

**Table 4:** Details of the 15 patients who underwent VF. A 〇 symbol is placed on the relevant one.

Age	Gender	Name of disease	FEES score	Length of hospital stay	Oral phase dysphagia	Esophageal dysphagia	Aspiration
69	Male	Postoperative gastric cancer	8	Less than 30 days		○	
27	Male	Multiple stroke	5	More than 180 days	○		
71	Male	None	8	More than 30 days but less than 90 days			
79	Male	Post radiotherapy for laryngeal cancer	5	More than 90 days but less than 180 days			○
67	Male	Post-operative tongue cancer	8	More than 180 days	○		○
85	Female	None	2	Less than 30 days			
77	Male	None	6	More than 180 days			○
83	Male	Postoperative gastric cancer	6	More than 180 days			○
72	Male	Ossification of anterior longitudinal ligament	6	More than 30 days but less than 90 days			○
71	Male	Amyloidosis	4	More than 180 days			○
65	Female	Functional dyspepsia	3	More than 30 days but less than 90 days		○	
87	Female	After covid-19 infection	8	More than 90 days but less than 180 days			○
63	Male	Postoperative thoracic aortic aneurysm	2	Less than 30 days			
84	Male	Alzheimer's dementia	8	More than 30 days but less than 90 days			○
70	Female	None	3	More than 30 days but less than 90 days			

## Discussion

The effectiveness of dysphagia outpatient rehabilitation by ear-nose-and-throat (ENT) clinics has received little attention. One report [5] found that, after more than one year of swallowing rehabilitation and dietary guidance in patients with pharyngeal symptoms and aspiration evident on swallowing endoscopy, sputum production decreased in 59%, sputum became less sticky in 24%, disappearance of choking in 18%, and 24% gained weight. In our previous study [6], a FEES Hyodo score ≥ 4 was found in all patients with swallowing disorders of the oral stage, or with oral dysphagia on swallowing; 40% improved after medical interview or assessment via FEES or VF. Another report [7] found that 17 of 33 severe cases who had undergone gastrostomy or experienced repeated bouts of pneumonia improved in terms of the Fujishima grade when outpatient swallowing and home care rehabilitation were combined, although treatment was sometimes discontinued because of death from old age or progression of underlying disease.

Overseas, rehabilitation tends to adhere to defined curricula; few cases have been reported. Standardised out-of-hospital management methods, including rehabilitation, assisted patients with Parkinson’s disease, particularly mild cases [8]. One report found that eight weeks of outpatient treatment was associated with a 78.8% improvement in treatment-resistant hypertension associated with obstructive sleep apnoea and dysphagia, as revealed by endoscopic swallowing [9]. Three-week outpatient treatment of patients with neuropathy after radiotherapy to treat mesopharyngeal cancer revealed improvements in all of the Mann Assessment of Swallowing Ability (MASA) score, the Functional Oral Intake Score (FOIS), the visual analogue scale (VAS) score, hyolaryngeal excursion, lingual-palatal pressures, and certain surface electromyographic findings; the effects persisted for up to three months [10].

In the present study, FEES revealed improvements in the TP and the extent of salivary pooling in the vallecular and piriform sinuses. Tongue activity is compromised by sarcopenia associated with dysphagia, even in healthy older adults; [11] the tongue plays an important role in moving food masses from the oral cavity to the pharynx and in pushing food along the pharyngeal wall into the oesophagus. A reduction in tongue strength may cause dysphagia involving both the oral and pharyngeal phases [12].

FEES is comparable to VF in terms of assessment of pharyngeal phase swallowing. Recent studies using the Hyodo scoring system showed that otolaryngologists could readily perform FEES and effectively predict aspiration; a Hyodo score ≥ 6 was the statistically strongest predictor of aspiration [13]. Some authors reported significant correlations between the hand grip strength and the peak expiratory flow rate; this aids evaluation of how strength training affects dysphagia [14]. In the present study, FEES revealed significant improvements in the extent of salivary pooling in the vallecular and piriform sinuses, indicating a reduction in subclinical aspiration.

Even when only weekly outpatient rehabilitation was scheduled, the TP revealed improvements in oral and pharyngeal swallowing, and FEES (which explores the pharyngeal phase) also indicated that treatment was effective. These results suggest that, in addition to current rehabilitation techniques, it may be necessary to consider other approaches, such as the use of interference wave stimulators [15] that improve the swallowing reflex by stimulating the superior laryngeal nerve.

In terms of contrast-enhanced swallowing, of the 15 patients for whom VF was performed, two exhibited disorders of the oral phase and two disorders of the oesophageal phase. As FEES is focused on the assessment of pharyngeal swallowing, the addition of VF to cases with suspected non-pharyngeal disorders, such as postoperative gastric or tongue cancer, rendered it possible to understand the pathologies of the oral, pharyngeal, and oesophageal phases. VF also guided treatment strategies in cases for whom prior treatment had been ineffective and who reported long disease courses.

The Guidelines for Dysphagia 2018 consider the indications for and limitations of outpatient rehabilitation in ENT clinics and discuss outpatient follow-up, swallowing instructions, referral to specialised medical institutions, and how to decide when further assessment and treatment are not required after the performance of FEES in ENT clinics [16]. In the present study, outpatient follow-up and swallowing instructions were sufficient. In addition, VF could be added to FESS, except in cases where surgery was needed to improve swallowing function or prevent aspiration. However, the ENT clinic alone could not handle cases adjudged to require more detailed assessment and dedicated treatment. Both house calls and collaboration with the home and nursing care sectors were required. These interactions require further study.

A limitation of this study is that it exclusively included relatively healthy participants who could visit the clinic, excluding patients with poor general health. Furthermore, the intervention was performed once a week; the consequences of increasing the frequency of intervention remain uncertain.

## Conclusions

Outpatient treatment at ENT clinics for patients who are able to maintain their ADLs to the extent that they are able to walk to a hospital is an option for the treatment of age-related dysphagia. For severe cases, however, house calls and collaboration with the home and nursing care sector will be necessary and should be considered in the future.
